# Flexible Gold-Based Electrochemical Biosensor for Highly Sensitive Detection of Saxitoxin in Water Samples

**DOI:** 10.1021/acsomega.5c11291

**Published:** 2026-03-06

**Authors:** Isadora Bernardes Sequalini, Thiago Teixeira da Silva, Lais Albuquerque Giraldi, Nirton Cristi Silva Vieira

**Affiliations:** † Institute of Science and Technology, 28105 Federal University of São Paulo, São José dos Campos 12231-280, Brazil; ‡ Detectacyn Ltda, Capivari 13363-260, Brazil

## Abstract

Saxitoxin (STX) is a highly potent cyanobacterial toxin, posing serious risks to aquatic ecosystems and human health. Conventional methods for STX detection, such as high-performance liquid chromatography (HPLC), mass spectrometry (MS), and enzyme-linked immunosorbent assays (ELISA), are effective but costly, labor-intensive, and nonportable, motivating alternative strategies. Here, we report a flexible electrochemical biosensor fabricated via photolithography of gold electrodes on affordable polymer-based substrates for STX detection in water samples. The electrodes showed high mechanical stability, low charge transfer resistance, and an electroactive surface area 55% larger than the geometric area. Anti-STX antibodies were immobilized using a simple sodium citrate-assisted physisorption method. The biosensor response, monitored by cyclic voltammetry in phosphate-buffered solution with [Fe­(CN)_6_]^3–/4–^ as the redox probe, displayed a concentration-dependent current decrease upon STX exposure. The biosensor operated within the range of 0.5–6.0 μg/L and showed a detection limit of 0.2 μg/L, which is 15 times lower than the World Health Organization (WHO) guideline value. The device exhibited high specificity, with negligible responses to microcystin-LR and cylindrospermopsin, and remained effective in mineral water samples across different pH values (6.2–10.2). This study highlights the potential of flexible, low-cost photolithographic sensors for practical monitoring of cyanotoxins in water samples, paving the way for broader applications in the detection of other environmentally relevant toxins.

## Graphical abstract

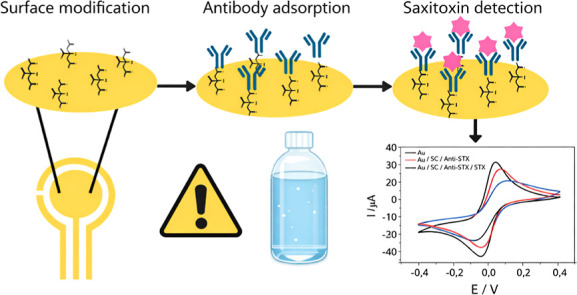

## 1 Introduction

Saxitoxin (STX) is recognized as one of the most potent naturally occurring neurotoxins produced by cyanobacteria and dinoflagellates during harmful algal blooms.^1^ Its occurrence poses a significant environmental and public health concern, as it contaminates marine and freshwater ecosystems and bioaccumulates in filter-feeding mollusks and other aquatic organisms.^1^ Human exposure occurs mainly through the ingestion of contaminated seafood or water, causing paralytic shellfish poisoning, a syndrome characterized by the blockade of voltage-gated sodium channels, which impairs nerve conduction and leads to paralysis, respiratory failure, and, in severe cases, death.^2^ More than 50 STX analogues, produced by different harmful algae, have been identified to date, including neosaxitoxin and gonyautoxins, with STX itself exhibiting the highest toxicity.^3^

Maximum allowable concentrations of STXs in drinking water differ among countries.^4^ In Brazil, the Ministry of Health has set a guideline value of 3.0 μg/L of STX equivalents for drinking water.^5^ STX equivalents represent the total saxitoxin toxicity expressed as the concentration of saxitoxin that would cause an equivalent toxic effect, considering the combined contribution of different STX analogues.^3^ To date, the World Health Organization (WHO) has adopted the same value as a health-based guideline for acute exposure, designed to protect infants and young children.^6^ However, this guideline may be updated in the future as new toxicological and epidemiological data become available.^6^

Traditional methods for STX detection include high-performance liquid chromatography (HPLC), mass spectrometry (MS), and enzyme-linked immunosorbent assays (ELISA). While effective, these methods are limited by high operational costs, labor-intensive procedures, and the need for specialized personnel, which can hinder their routine use in large-scale environmental monitoring and resource-limited settings.^7^

To overcome the limitations of traditional methods, biosensors have emerged as rapid and cost-effective tools for the detection of cyanotoxins, including STX.^8^ Among these, electrochemical biosensors have become the most widely employed. For example, potentiometric biosensors using lipid films functionalized with antisaxitoxin (anti-STX) antibodies on graphene-modified electrodes have been developed.^9^ Similarly, potentiometric devices employing anti-STX aptamers immobilized via electrostatic interactions on a poly­(allylamine hydrochloride) layer have also been reported.^10^ Both devices demonstrated good sensitivity and satisfactory performance for the detection of STX in spiked samples, including mussels and lake water.^9,10^

Impedimetric biosensors have also been applied for the detection of STX. Serrano et al. used a conventional three-electrode electrochemical cell with an aptamer-modified gold electrode, achieving a limit of detection (LOD) of 0.3 μg/L and high specificity for STX.^11^ In contrast, Wang et al. developed a compact, integrated sensor using interdigitated gold electrodes modified with cardiomyocytes as the recognition system, detecting STX-induced changes in sodium channels in cell membranes with a LOD of 0.5 μg/L.^12^

Voltammetric biosensors can also be highlighted. Liu et al. reported a conventional three-electrode system with a glassy carbon electrode modified by gold nanoparticles and reduced graphene oxide, further functionalized with a selective peptide for STX, enabling detection at ultralow levels (0.00069 μg/L) using differential pulse voltammetry.^13^ On the other hand, Rhouati et al. developed a multiplexed platform with eight carbon-printed electrodes, each modified with gold nanoparticles and aptamers targeting five cyanotoxins. Using square wave voltammetry, the biosensor detected STX through aptamer conformational changes, achieving a LOD of 0.0053 nM (i.e., 0.0016 μg/L) in water samples.^14^

The miniaturization of electrochemical systems is widely recognized as a key advance in biosensor development, as it enhances portability and integration, enables rapid on-site analysis with small sample volumes, improves sensitivity, and enables cost-effective, large-scale fabrication.^15^ Such systems are typically produced using microfabrication techniques, including screen printing, inkjet printing, and even 3D printing of conductive materials, such as gold, silver, carbon materials, or a combination of them.^15^

While some microfabrication techniques are practical and economical, photolithography provides superior resolution and reproducibility for demanding electrochemical applications.^16^ Although more costly, this technique enables the precise patterning of electrodes at the micrometer scale, allowing for complex multilayer designs that are difficult to achieve with printing methods. Its exceptional control over feature size and geometry makes photolithography particularly valuable for research applications and high-performance, reproducible electrochemical systems where precision is required.^17^

In addition, photolithography is compatible with flexible substrates, which generally cost less, enabling precise patterning on unconventional substrates while maintaining performance under mechanical stress. Oliveira et al. demonstrated this capability by fabricating flexible platinum electrodes on biobased polyethylene terephthalate (Bio-PET) substrates via photolithography.^18^ Their electrodes achieved 50 μm feature resolution and retained functionality after repeated bending, with no significant change in electrochemical response. The devices successfully detected Parkinson’s disease biomarkers (dopamine and PARK7/DJ-1 protein) at clinically relevant concentrations, demonstrating the suitability of photolithography for high-performance flexible biosensors.^18^ Recently, we demonstrated the fabrication of interdigitated gold electrodes using photolithography on low-cost pouch film substrates, which are traditionally used for document binding. This approach enabled precise patterning of gold electrodes (100 μm features) with excellent mechanical properties.^19^

Here, we show the development of a compact electrochemical biosensor for the detection of STX in water samples, fabricated by photolithography of gold electrodes on low-cost flexible substrates. Unlike most reported electrochemical biosensors, which depend on bulky three-electrode cells or require multiple steps of nanomaterial modification and sophisticated recognition systems, our device relies on a single functionalization step, simplifying its preparation and use. Cyclic voltammetry (CV) and electrochemical impedance spectroscopy (EIS) analysis confirmed consistent electrode performance using a [Fe­(CN)_6_]^3–/4–^ redox probe. The biosensor achieved a LOD of 0.2 μg/L for STX, i.e., 15 times lower than the WHO guideline limit, and showed no response for other cyanotoxins. In addition, the biosensor performed reliably in different mineral water samples spiked with STX, thereby confirming its practical applicability for environmental monitoring under near-real conditions.

## 2 Experimental Section

### 2.1 Materials

Phosphate-buffered saline (PBS), potassium hexacyanoferrate (III), and potassium hexacyanoferrate (II) trihydrate were acquired from Sigma-Aldrich. Sodium citrate tribasic dihydrate was purchased from Synth (Brazil). STX standard was supplied by the Cawthron Institute (New Zealand). Polyclonal antisaxitoxin (anti-STX) antibodies (catalog number AS11 1647) were obtained from Agrisera (Sweden). Ultrapure water (Milli-Q, resistivity ≥18.2 MΩ·cm) was used for the preparation of all solutions. Commercial mineral water samples with different nominal pH values (also confirmed using a pH meter) were obtained from local vendors for matrix interference studies.

### 2.2 Microfabrication and Characterization of Gold Electrodes

The pouch film substrates (125 μm thick) consisted of a multilayer structure of polyethylene terephthalate/polyethylene/ethylene-vinyl acetate (PET/PE/EVA) and were obtained from Spiral Brazil. A three-electrode electrochemical system was fabricated on the PET surface by photolithography using a dark-field mask, at the facilities of the Microfabrication and Microfluidics Laboratory of the Brazilian Nanotechnology National Laboratory (LNNano), following procedures described previously.^19^ Briefly, the substrates containing the electrode pattern underwent sputtering to deposit a 20 nm chromium layer (to improve adhesion), followed by a 120 nm gold layer to establish the conductive traces. Figure 1a shows a photograph of the fabricated electrodes, and Figure 1b illustrates how an electrode was used during electrochemical characterization. The working (W), counter (C), and reference (R) electrodes have areas of 7.0, 8.3, and 2.3 mm^2^, respectively.

**Figure 1:**
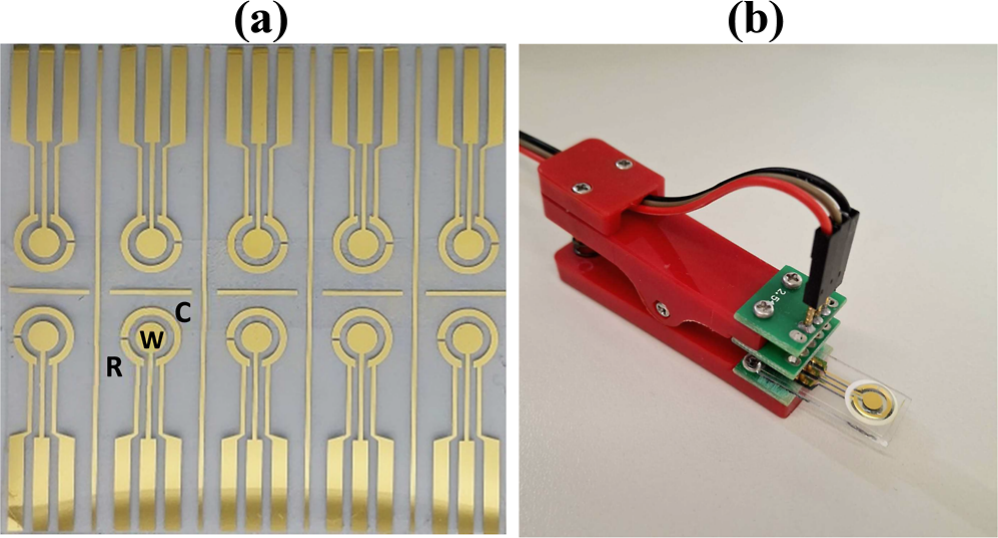
(a) Photograph of the fabricated interdigitated gold electrodes, with R, W, and C denoting the reference, working, and counter electrodes, respectively. (b) Electrochemical characterization of an electrode. An adhesive layer was used to delimit the analytical solution.

The electrodes were analyzed using scanning electron microscopy (SEM) combined with energy-dispersive X-ray spectroscopy (EDS) on a FEI Inspect S50 microscope and by X-ray diffraction (XRD) with a Rigaku Ultima IV diffractometer.

Electrochemical characterization (CV and EIS) was performed using a PalmSens4 potentiostat/galvanostat in PBS containing an equimolar mixture of 1.0 mmol/L [Fe­(CN)_6_]^3–/4–^ as the redox probe to evaluate the reproducibility of the electrodes.

The electroactive surface area (ESA) of the gold working electrode was determined based on the adsorption of oxygen on the gold surface in an acidic medium. In this case, cyclic voltammograms were recorded in 0.1 mol/L H_2_SO_4_ within a potential range of −0.2 to 0.8 V at a scan rate of 100 mV/s. ESA was calculated using the following equation

$$ESA=\left(Q_{red}-Q_{dl}\right)/\theta^{o}\times Q^{AuO}$$

where *Q*_red_ represents the charge obtained by integrating the cathodic peak corresponding to gold oxide reduction, and *Q*_dl_ corresponds to the charge associated with the double-layer capacitance, θ^0^ denotes the fractional surface coverage by gold oxide, and *Q* ^AuO^ is the charge required to reduce a monolayer of gold oxide per unit area, reported as 390 μC/cm^2^.^20^

### 2.3 Biosensor Preparation

Before immobilizing the antibodies, the electrodes were cleaned in an ultrasound bath in acetone, isopropyl alcohol, and ethyl alcohol. They were then washed with deionized water and dried at room temperature. Next, 20 μL of a 100 mmol/L sodium citrate solution were deposited onto the working electrode for 30 min. Then, an anti-STX antibody solution (40 μg/L) prepared in PBS buffer was dispensed onto the electrode surface and left incubated for 1 h under humid conditions to ensure proper immobilization.^21^

### 2.4 Sample Preparation and Electrochemical Measurements

The commercial STX standard (10 μg) was diluted in an aqueous solution of 100 mmol/L acetic acid to obtain a stock concentration of 10 μg/mL. Aliquots at the desired concentrations were subsequently prepared in ultrapure water. Before depositing onto the working electrode, the samples were diluted 1:1 in PBS to prevent antibody denaturation. Thus, the effective concentration analyzed corresponded to half the nominal value. Control (blank) samples were prepared under identical conditions, containing the same proportion of diluted acetic acid as the STX solutions.

For the mineral water samples analysis, three commercially available mineral waters from different manufacturers, each with different nominal pH values, were purchased from local vendors. Each mineral water sample was spiked with STX at a 3 μg/L concentration and diluted in PBS (1:1) before the electrochemical analyses.

CV measurements were performed before and after incubating 20 μL of the prepared STX solution (previously diluted 1:1 in PBS) on the working electrode for 60 min, followed by a gentle PBS rinse to remove unbound molecules. Electrochemical measurements were performed in PBS containing an equimolar mixture of 1.0 mmol/L [Fe­(CN)_6_]^3–/4–^ as the redox probe.

All potentials in the cyclic voltammograms were referenced to the gold pseudoreference electrode, and all experiments were performed at room temperature (25 ± 1 °C).

## 3 Results and Discussion

### 3.1 Characterization of the Gold Electrodes

Figure 2a shows the SEM micrograph (10,000× magnification) of the surface of one of the fabricated electrodes. Some imperfections, such as localized gold accumulation forming small metallic islands and occasional surface defects, can be observed. These imperfections are typical of microfabrication when substrates with inherent surface irregularities are employed. Nevertheless, such variations are expected and acceptable as long as they do not compromise the functionality of the electrodes. The lack of uniformity among electrodes fabricated under the same conditions may affect the reproducibility of the electrochemical responses, which will be reflected in the standard deviation of the electrochemical measurements. Figure 2b shows the EDS spectrum and elemental mapping, confirming the presence of C (carbon) from the substrate and Au (gold) and Cr (chromium) from the metallic layers, with no undesired elements detected.

**Figure 2:**
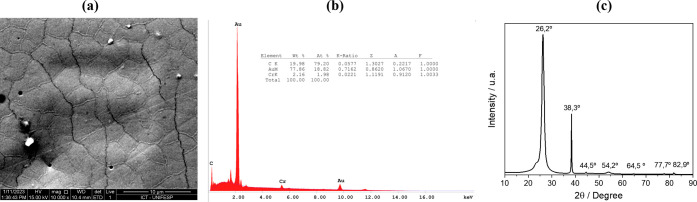
(a) SEM micrograph of the gold electrode surface at 10,000× magnification. (b) EDS spectrum and elemental mapping showing the spatial distribution of chemical elements across the electrode surface. (c) XRD pattern of the Au/Cr/PET.

Figure 2c shows the XRD pattern of the electrode. The diffraction peak at 26.2° corresponds to PET, which can exhibit amorphous or semicrystalline phases with diffraction around 26°.^22^ The EVA polymer can also present a semicrystalline phase, with diffraction near 21°.^23^ Since the manufacturer does not specify the layer orientation (the pouch film consists of PET–PE-EVA polymers), we conclude that the metallic films were deposited on the PET side. Additionally, the diffraction peak at 54.2° can be attributed to the PET substrate, as reported previously.^24,25^ The diffraction peaks at 38.3°, 44.5°, 64.5°, 77.7°, and 82.9° correspond to the (111), (200), (220), (311), and (222) planes of the face-centered cubic gold structure (JCPDS: 04–0784).^26^ Besides the dominant (111) plane, the other characteristic gold peaks are partially masked by the PET diffraction peaks observed in Figure 2c.

CV and EIS techniques were employed to evaluate the reproducibility of the electrodes. To this end, 50 μL of a 0.1 M PBS solution containing 1.0 mmol/L of the redox probe [Fe­(CN)_6_]^3–/4–^ was directly deposited onto the electrode. Figure 3a,b show the cyclic voltammograms and Nyquist plots for five electrodes obtained from different substrates. Well-defined anodic (*E*_pa_) and cathodic (*E*_pc_) peaks were observed at approximately +0.026 V and −0.060 V, respectively. The corresponding anodic (*I*_pa_) and cathodic (*I*_pc_) peak currents were found to be (32.8 ± 0.7) μA and (−30.8 ± 0.6) μA. The calculated peak-to-peak separation (Δ_Ep_) was (0.083 ± 0.001) V, and the *I*_pc_/*I*_pa_ ratio was 0.94. These results indicate well-defined redox behavior with moderate peak separation under the experimental conditions employed. Using the integrated area under the voltammograms as a comparison parameter, an average area value of (10.6 ± 0.2) μA·V was obtained, corresponding to a relative standard deviation (RSD) of 1.9%. This excellent interelectrode reproducibility is mainly attributed to the microfabrication process, which ensures high uniformity of electrode geometry, as well as to the controlled and reproducible cleaning protocol.

**Figure 3:**
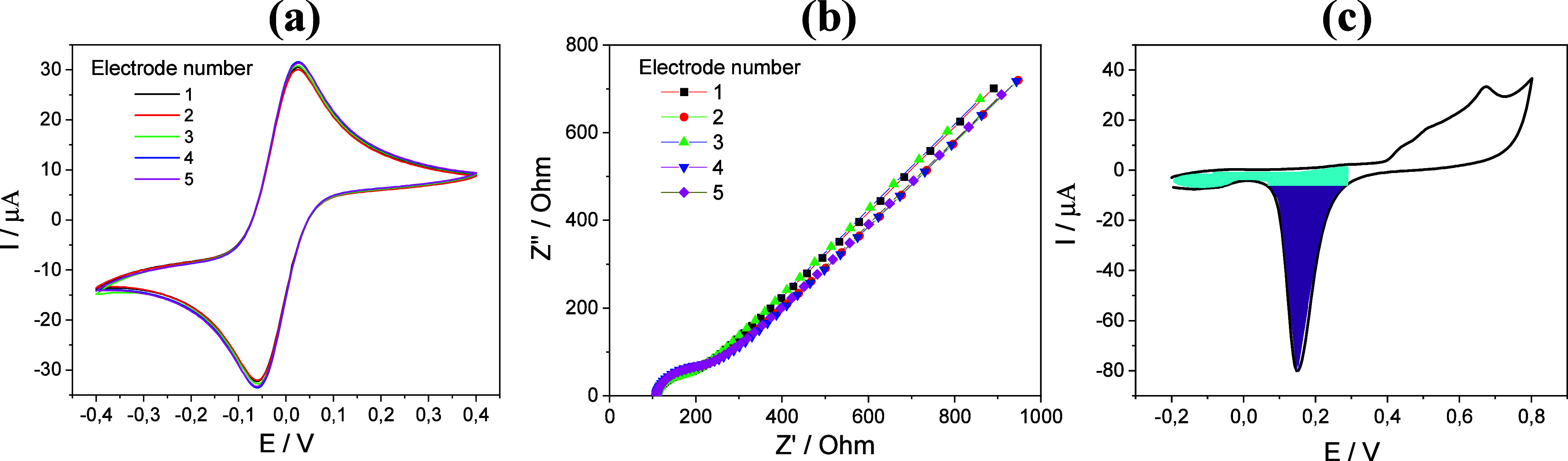
(a) Cyclic voltammograms and (b) Nyquist plots obtained from five gold electrodes fabricated on different substrates. Measurements were performed in PBS containing 1.0 mmol/L [Fe­(CN)_6_]^3–/–^ at a scan rate of 100 mV/s. (c) Cyclic voltammogram of the gold electrode recorded in 0.1 mol/L H_2_SO_4_ at a scan rate of 100 mV/s. The shaded purple region represents the charge associated with gold oxide reduction, and the shaded blue region represents the double-layer charge.

The Nyquist plots (Figure 3b) show that the electrodes exhibit a very low charge-transfer resistance (*R*_ct_). This indicates a fast electron-transfer process at the electrode–electrolyte interface, which is highly desirable for biosensors.^27^ Furthermore, by fitting the impedance data to a Randles equivalent circuit using PSTrace software, an average *R*_ct_ of (226.3 ± 2) Ω was obtained. The CV and EIS results confirm the high reproducibility and excellent electrochemical performance of the electrodes, reinforcing their potential for reliable use in sensing platforms.

Figure 3c shows the cyclic voltammogram recorded in 0.1 mol/L H_2_SO_4_ for the ESA determination. The ESA of the fabricated electrodes was found to be (10.9 ± 0.2) mm^2^, approximately 55% greater than their geometric area. This increase reflects the microscopic roughness and structural features of the gold surface, which enhance the effective area available for electrochemical reactions.

The electrochemical behavior of our electrodes is consistent with previous reports on noble-metal electrodes deposited on flexible substrates or even superior. For example, Oliveira et al. fabricated platinum electrodes on BioPET by lithography, which showed well-defined redox peaks with a Δ_Ep_ of 0.92 mV, an *I*_pc_/*I*_pa_ of 1.0, and a remarkably high ESA of 0.28 cm^2^ compared to a geometric area of 0.03 cm^2^. However, in contrast to our approach, ESA was determined using a redox probe of 0.1 mol/L KCl and 1.0 mmol/L [Fe­(CN)_6_]^3–/4––^, and using the Randles–Sevick equation rather than by direct reduction of metallic oxides.^18^ Cavalhal et al. fabricated titanium/gold electrodes (20/100 nm) by lithography on polyester films to develop enzymatic biosensors, reporting Δ_Ep_ values dependent on the geometric area, ranging from ca. 0.09 to 0.29 V for areas of 3.0 mm^2^ and 0.30 mm^2^, respectively. The electroactive areas were about 2.5 times greater than the geometric areas, and the *I*_pc_/*I*_pa_ ratios were close to 1.^28^ On the other hand, Faria and Zucolotto produced gold electrodes (150 nm) deposited directly onto PET by sputtering using a metal mask, which exhibited Δ_Ep_ of (0.097 ± 0.010) V, *I*_pc_/*I*_pa_ ratio of 1.04 ± 0.20, and a *R*_ct_ of (102 ± 10) Ω.^29^

### 3.2 STX Biosensing with Gold Electrodes

Before proceeding with the detection experiments, CV was used to monitor the immobilization of anti-STX antibodies on the working electrode. Figure 4a shows the cyclic voltammograms recorded for the bare electrode (black curve) and after its modification with anti-STX immobilization on citrate-modified gold electrodes (red curve). As expected, antibody immobilization hinders the charge transfer between the redox probe and the electrode surface, as evidenced by the reduced voltammogram area and decreased peak currents after functionalization. In addition, the slight peak shifting observed in the voltammograms is consistent with slower electron transfer kinetics, resulting from the formation of an insulating biorecognition layer at the electrode interface.

**Figure 4:**
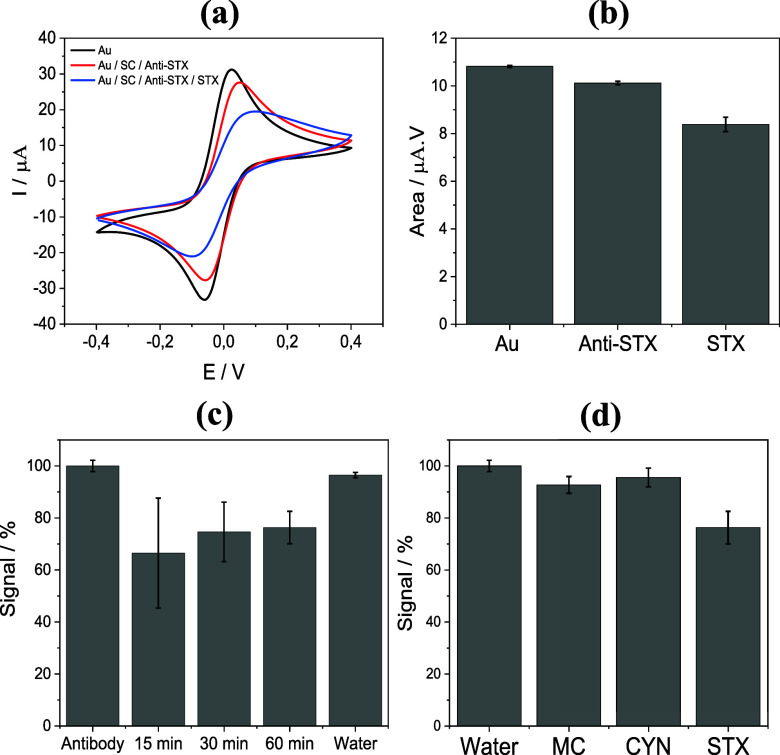
(a) Cyclic voltammograms obtained for the bare gold electrode, after modification with anti-STX antibodies, and following the detection of 3 μg/L STX. Measurements were performed in PBS containing 1.0 mmol/L [Fe­(CN)_6_]^3–/4–^ at a scan rate of 100 mV/s. (b) Signal areas for each condition, obtained from the integrated area of the entire cyclic voltammogram. (c) Percentage signals obtained at different incubation times for the detection of 3 μg/L STX. (d) Percentage signals obtained for control and specificity tests: no STX (water control), 1.0 μg/L microcystin-LR (MC), 1.0 μg/L cylindrospermopsin (CYN), and 3 μg/L STX. Error bars indicate standard deviation calculated from five independent measurements (*n* = 5), except for selectivity assays (*n* = 3).

In the immobilization strategy adopted in this study, antibodies were weakly bound to the gold electrode surface through hydrophobic and electrostatic interactions. The advantages of this strategy include the use of low-cost reagents, a single immobilization step, and high reproducibility. Kim et al. challenged the prevailing assumption that covalent immobilization via NHS/EDC chemistry (widely employed in biosensors) is inherently superior, demonstrating that physisorption can be a more efficient, cost-effective, and scalable alternative for antibody immobilization on gold surfaces, while also minimizing unwanted interactions with other biomolecules.^21^

Upon interaction with STX, the modified surface became even more resistive, further decreasing the voltammetric response (Figure 4a, blue curve). Considering the voltammogram area, which is proportional to the charge transferred during the redox process, as 100% of the initial signal for the bare electrode, a decrease of approximately 10% was observed after antibody immobilization, and about 25% after STX detection. More specifically, for *n* = 5, the normalized signal areas were (100 ± 2) % for the bare electrode, (91 ± 3) % after anti-STX immobilization, and (75 ± 6) % following STX exposure, as indicated in the bar graph of Figure 4b.

To optimize the detection time of the assay, the response to 3.0 μg/L STX was evaluated at different incubation times, followed by CV analysis. The results are shown in Figure 4c. STX was detected at all tested times (15, 30, and 60 min). However, the reproducibility was lower at 15 and 30 min, with signal of (67 ± 21) % and (75 ± 11) %, respectively, compared to the value of (76 ± 6) % at 60 min. This reduced reproducibility may be due to incomplete binding equilibrium between the antibody and STX at relatively shorter incubation times. The signal obtained for the water sample without STX (negative control) was (96 ± 1) %, slightly lower than that of the antibody-modified electrode (100 ± 2) %, which was taken as the reference (set to 100%). This slight decrease may be attributed to variations during the incubation and washing steps. Based on the improved reproducibility and lower uncertainty, an incubation time of 60 min was selected for subsequent assays.

For specificity testing of the biosensor, microcystin-LR (MC) at 1.0 μg/L and cylindrospermopsin (CYN) at 1.0 μg/L were used as nontarget analytes. These toxin concentrations were selected based on the maximum allowable limits for drinking water, as recommended by the WHO.^30^ Water was again used as the negative control. Figure 4d shows that the signals obtained for MC and CYN were negligible, closely matching the water control and clearly distinct from the STX (3.0 μg/L) signal, indicating no significant cross-reactivity. This close similarity confirms the absence of nonspecific binding and further supports the high specificity of our biosensor for STX.

To construct the analytical curve, STX concentrations were varied from 0 (water control), 0.5, 1.0, 2.0, 3.0, and 6.0 μg/L. Figure 5a shows the average responses obtained from five independent replicates for each concentration. The biosensor was able to clearly differentiate positive from negative samples at concentrations as low as 0.5 μg/L, demonstrating reliable discrimination performance. The LOD was calculated as 0.2 μg/L using the standard relation LOD = (3σ*b*)/*S*, where σ*b* represents the standard deviation of the blank, and *S* is the slope of the analytical curve.^31,32^ This low LOD highlights the excellent performance of the flexible gold electrode and the efficiency of the immobilization and detection strategy employed in this study. Importantly, the obtained LOD is 15 times lower than the maximum concentration recommended by the WHO for STX in drinking water, underscoring the potential of our biosensor for environmental monitoring and public health applications.

**Figure 5:**
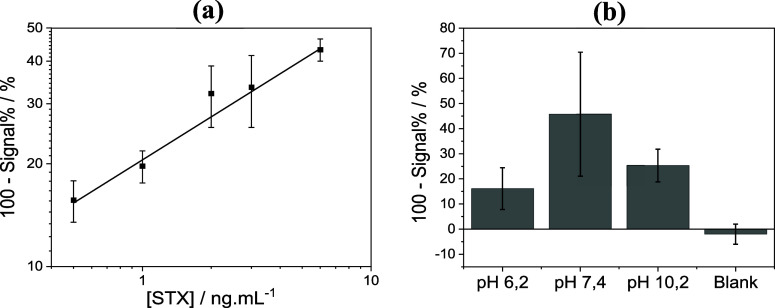
(a) Analytical curve obtained for water samples spiked with STX at concentrations from 0 to 6.0 μg/L. (b) Response for mineral water samples spiked with 3.0 μg/L STX at pH 6.2, 7.4, and 10.2. Error bars indicate standard deviation calculated from five independent measurements (*n* = 5).

Finally, tests were conducted using mineral water samples with nominal pH values of 6.2, 7.4, and 10.2. Each mineral water sample was spiked with STX at a concentration of 3.0 μg/L. The results are shown in Figure 5b. Taking the signal corresponding to a concentration of 3.0 μg/L in the analytical curve, an apparent recovery of 51%, 135%, and 77% for pH values of 6.2, 7.4, and 10.2, respectively, was found. During the incubation step, the samples were prepared in PBS mixed at a 1:1 (v/v) ratio with STX-spiked water samples (see Experimental Section). Under these conditions, the buffering capacity of PBS is expected to maintain the pH relatively stable throughout the incubation process. The pH values measured after mixing mineral water samples with PBS were 7.5, 7.5, and 8.1 for the samples initially at pH 6.2, 7.4, and 10.2, respectively. Therefore, no significant pH changes occurred under the experimental conditions employed. Thus, the signal variations observed among the mineral water samples may be due to differences in ionic composition, not just pH variations. Despite the relatively high variability, as evidenced by the error bars in Figure 5b, the biosensor was able to distinguish the spiked (“contaminated”) samples from the blank (no STX) signal. These findings indicate that the biosensor maintains its detection capability even in different matrices. Further optimizations could improve response consistency under varying environmental conditions, such as changes in temperature, pH, or sample matrix composition. However, these aspects are beyond the scope and objectives of the present study. Overall, the results demonstrate the performance of the proposed biosensor and highlight its potential for reliable STX detection in different samples.

Table 1 compares the analytical performance of our biosensor with previously reported electrochemical and electrical sensors for STX detection. Although some nanomaterial or peptide-based sensors achieve lower LOD values, they often rely on more complex fabrication strategies, such as semiconductor-based architectures,^10^ combination of nanomaterials,^13^ or cell-based platforms.^12^ Overall, the proposed biosensor provides a well-balanced combination of low LOD, analytical performance, and experimental simplicity for STX detection. The excellent performance of the biosensor can be mainly attributed to the high electrical conductivity of the gold electrode and to the surface modification achieved via sodium citrate adsorption, which promoted a stable and homogeneous functional layer. This strategy favored effective antibody immobilization while preserving the electroactive surface, resulting in fast electron-transfer kinetics and low interfacial charge-transfer resistance. The optimized surface chemistry and the stable electrochemical response directly contributed to the good performance of the device.

**Table 1:** Comparison of Analytical Performance of Different Biosensors for STX Detection^a^

electrode	recognition element	technique	operating range (μg/L)	LOD (μg/L)	recovery (%)	RSD (%)	reference
Copper-supported graphene coated with a polymerized lipid film	Anti-STX antibody	Potentiometric	0.29–29.9	0.29	90–106	NR	^9^
*n*Si/SiO_2_ poly(allylamine hydrochloride) layer	Aptamer	Potentiometric (electrolyte-insulator-semiconductor)	0.15–29.9	0.015	111–116	NR	^10^
Bare gold electrode	Aptamer	Electrochemical impedance spectroscopy	0.3–30	0.3	NR	NR	^11^
Gold interdigitated electrode integrated into a multiwell platform	Living cells (cardiomyocytes)	Electrical impedance	0.69–1.26	0.087	NR	NR	^12^
Glassy carbon electrode modified with reduced graphene oxide/gold nanoparticles	Peptide	Differential pulse voltammetry	0.001–1.0	0.00069	87.3–116.2	3.4	^13^
WHO guideline (drinking water)	-	-	-	3.0	-	-	^33^
This work	Anti-STX antibody	Cyclic voltammetry	0.5–6.0	0.2	0.18	1.9	This study

a NR = not reported.

## 4 Conclusions

This study demonstrates the successful development of a flexible electrochemical biosensor that combines low-cost fabrication, simple functionalization, and reliable analytical performance for the detection of STX in water samples. The device comprised photolithographically patterned gold electrodes on pouch film substrates, resulting in mechanically stable and electrochemically efficient sensors. The use of a single-step, sodium citrate-assisted antibody immobilization strategy not only simplifies preparation but also reduces costs, highlighting the practicality of this approach for large-scale implementation. Beyond the excellent selectivity and low detection capability achieved, the biosensor showed consistent performance across different pH conditions in mineral water samples, reinforcing its applicability in near-real scenarios. These findings underscore the potential of this platform to support monitoring of STX in aquatic systems and open new perspectives for adapting similar strategies to detect a broader range of environmentally and clinically relevant toxins.

## References

[1] Deng H., Shang X., Zhu H., Huang N., Wang L., Sun M. S. (2025). A Comprehensive Review of Its History, Structure, Toxicology,
Biosynthesis, Detection, and Preventive Implications. Mar. Drugs.

[2] Lee J., Lee S., Jiang X. (2017). Cyanobacterial
Toxins in Freshwater and Food: Important
Sources of Exposure to Humans. Annu. Rev. Food
Sci. Technol..

[3] Ballot, A. , Bernard, C. , Saxitoxin, J. F. , Analogues Handbook of Cyanobacterial Monitoring and Cyanotoxin Analysis 2016, 148–154.

[4] Cotruvo J.
A. (2022). Algal Toxins
in Drinking Water: Standards and Guidelines. J. AWWA.

[5] Santos-Silva R. D. d., Severiano J. d. S., Chia M. A., Queiroz T. M., Cordeiro-Araújo M. K., Barbosa J. E. d. L. (2024). Unveiling
the Link between Raphidiopsis Raciborskii Blooms and Saxitoxin Levels:
Evaluating Water Quality in Tropical Reservoirs, Brazil. Environ. Pollut..

[6] Organization, W. H. Cyanobacterial Toxins: Saxitoxins; World Health Organization, 2020.

[7] Ishak S. M., Yahaya N., Loh S. H., Kamaruzaman S., Zain N. N. M., Waras M. N., Abdullah W. N. W., Miskam M., Raoov M., Aziz N. A. (2023). Research
Progress on
Extraction and Analytical Methods for Saxitoxin and Its Congeners. Chromatographia.

[8] Vogiazi V., de la Cruz A., Mishra S., Shanov V., Heineman W. R., Dionysiou D. D. (2019). A Comprehensive
Review: Development of Electrochemical
Biosensors for Detection of Cyanotoxins in Freshwater. ACS Sens..

[9] Bratakou S., Nikoleli G., Siontorou C. G., Nikolelis D. P., Karapetis S., Tzamtzis N. (2017). Development of an Electrochemical
Biosensor for the Rapid Detection of Saxitoxin Based on Air Stable
Lipid Films with Incorporated Anti-STX Using Graphene Electrodes. Electroanalysis.

[10] Noureen B., Ullah N., Tian Y., Du L., Chen W., Wu C., Wang P. (2022). An Electrochemical PAH-Modified Aptasensor for the
Label-Free and Highly-Sensitive Detection of Saxitoxin. Talanta.

[11] Serrano P. C., Nunes G. E., Avila L. B., Reis C. P. S., Gomes A. M. C., Reis F. T., Sartorelli M. L., Melegari S. P., Matias W. G., Bechtold I. H. (2021). Electrochemical
Impedance Biosensor for Detection of Saxitoxin in Aqueous Solution. Anal. Bioanal. Chem..

[12] Wang Q., Su K., Hu L., Zou L., Wang T., Zhuang L., Hu N., Wang P. (2015). A Novel and
Functional Assay for Pharmacological Effects
of Marine Toxins, Saxitoxin and Tetrodotoxin by Cardiomyocyte-Based
Impedance Biosensor. Sens. Actuators, B Chem..

[13] Liu B., Chen L., Zhu Y., Zhao X., Wang H., Wang S. (2024). A Novel Screening on
the Specific Peptides by Molecular Simulation
and Development of the Highly-Sensitive Electrochemical Sensor for
Saxitoxin. Microchem. J..

[14] Rhouati A., Zourob M. (2024). Development of a Multiplexed Electrochemical Aptasensor
for the Detection of Cyanotoxins. Biosensors.

[15] Wu J., Liu H., Chen W., Ma B., Ju H. (2023). Device Integration
of Electrochemical Biosensors. Nat. Rev. Bioeng..

[16] Fruncillo S., Su X., Liu H., Wong L. S. (2021). Lithographic Processes for the Scalable
Fabrication of Micro-and Nanostructures for Biochips and Biosensors. ACS Sens..

[17] Ayres L. B., Pimentel G. J. C., Costa J. N. Y., Piazzetta M. H. O., Gobbi A. L., Shimizu F. M., Garcia C. D., Lima R. S. (2024). Ultradense
Array of On-Chip Sensors for High-Throughput Electrochemical Analyses. ACS Sens..

[18] Oliveira G. C. M. d. G. C. M. D., Carvalho J. H., Brazaca L. C., Vieira N. C. S., Janegitz B. C., Janegitz B. C. B. C. (2020). Flexible Platinum
Electrodes as Electrochemical
Sensor and Immunosensor for Parkinson’s Disease Biomarkers. Biosens. Bioelectron..

[19] Braga T. S., Sequalini I. B., da Silva T. T., Marcellino G. M., Corat E. J., Vieira N. C. S. (2025). Electrochemical
Reduction of Graphene
Oxide on Flexible Interdigitated Electrodes and Its Application as
Strain Sensors. Phys. Status Solidi A.

[20] Lukaszewski M., Soszko M., Czerwiński A. (2016). Electrochemical
Methods of Real Surface
Area Determination of Noble Metal Electrodes - an Overview. Int. J. Electrochem. Sci..

[21] Kim K., Son T., Hong J.-S., Kwak T. J., Jeong M. H., Weissleder R., Im H. (2022). Physisorption of Affinity Ligands Facilitates Extracellular Vesicle
Detection with Low Non-Specific Binding to Plasmonic Gold Substrates. ACS Appl. Mater. Interfaces.

[22] Johnson J. E. (1959). X-ray Diffraction
Studies of the Crystallinity in Polyethylene Terephthalate. J. Appl. Polym. Sci..

[23] Xu S., Li J., Ye Q., Shen L., Lin H. (2021). Flame-Retardant
Ethylene
Vinyl Acetate Composite Materials by Combining Additions of Aluminum
Hydroxide and Melamine Cyanurate: Preparation and Characteristic Evaluations. J. Colloid Interface Sci..

[24] Sabry R. S., Kammel R. S. (2018). Flexible Sandwich Piezoelectric Nanogenerators
Based
ZnO Nanorods for Mechanical Energy Harvesting. Al-Mustansiriyah J. Sci..

[25] Faraj M. G., Eisa M. H., Pakhuruddin M. Z. (2019). Physical
Properties of Spray Pyrolysed
Cadmium Sulfide Thin Films Deposited on Different Polymer Substrates. Int. J. Electrochem. Sci..

[26] Bulowski W., Skibińska K., Żabiński P., Wojnicki M. (2025). Optimization
of Gold Thin Films by DC Magnetron Sputtering: Structure, Morphology,
and Conductivity. Coatings.

[27] Figueiredo A., Vieira N. C. S., Dos Santos J. F., Janegitz B. C., Aoki S. M., Junior P. P., Lovato R. L., Nogueira M. L., Zucolotto V., Guimaraes F. E. G. (2015). Electrical
Detection of Dengue Biomarker Using Egg
Yolk Immunoglobulin as the Biological Recognition Element. Sci. Rep..

[28] Carvalhal R. F., Machado D. S., Mendes R. K., Almeida A. L. J., Moreira N. H., Piazetta M. H. O., Gobbi A. L., Kubota L. T. (2010). Development of a
Disposable Amperometric Biosensor for Salicylate Based on a Plastic
Electrochemical Microcell. Biosens. Bioelectron..

[29] Faria H. A. M., Zucolotto V. (2019). Label-Free
Electrochemical DNA Biosensor for Zika Virus
Identification. Biosens. Bioelectron..

[30] Ishak S. M., Yahaya N., Loh S. H., Kamaruzaman S., Zain N. N. M., Waras M. N., Abdullah W. N. W., Miskam M., Raoov M., Aziz N. A. (2023). Research
Progress on
Extraction and Analytical Methods for Saxitoxin and Its Congeners. Chromatographia.

[31] Currie L. A. (1968). Limits
for Qualitative Detection and Quantitative Determination. Application
to Radiochemistry. Anal. Chem..

[32] Long G. L., Winefordner J. D. (1983). Limit of
Detection. A Closer Look at the IUPAC Definition. Anal. Chem..

[33] Organization, W. H. Guidelines for Drinking-Water Quality, 4 ed.; World Health Organization: Geneva, 2017.

